# Investigating Disparities in Metabolite Concentrations Using Gas Chromatography Mass Spectroscopy Among Patients with Alzheimer's Disease and Healthy Subjects

**DOI:** 10.1002/alz70857_105349

**Published:** 2025-12-25

**Authors:** Ravinder Singh, Pratima Singh, Suruchi Chaubey

**Affiliations:** ^1^ Chitkara University, Rajpura, Punjab, India

## Abstract

**Background:**

A useful technique for comprehending the intricate metabolic changes linked to neurodegenerative diseases like Alzheimer's disease (AD) is metabolomics. This study used gas chromatography‐mass spectrometry (GC‐MS) to investigate metabolic variations between Alzheimer's patients and healthy controls, with an emphasis on amino acid metabolism and its potential relation to cognitive function decrease.

**Method:**

A total of 120 participants were recruited and split into two groups: healthy controls (Group A) and AD sufferers (Group B). To assess the amounts of amino acid metabolites, GC‐MS was used to examine plasma samples from both groups. Memory, executive function, speed of thinking, and attention were all evaluated using established neuropsychological tests.

**Result:**

The levels of tryptophan (35.62±6.32 μM), methionine (18.75±5.21 μM), phenylalanine (45.12±5.08 μM), and tyrosine (50.87±5.76 μM) were all much lower in AD patients than in healthy controls. On the other hand, AD patients had higher levels of aspartate (23.57±4.10 μM) and glycine (280.43±7.15 μM). Cognitive problems such as memory loss, slowed processing speed, and poor executive function were linked to these metabolic changes.

**Conclusion:**

According to our research, Alzheimer's disease is linked to elevated levels of glycine and aspartate and decreased levels of phenylalanine, tyrosine, methionine, and tryptophan. These amino acid disturbances are associated with cognitive deterioration, implying that they might be used as biomarkers for Alzheimer's disease diagnosis and treatment. Supplementing with amino acids may be a viable strategy to slow the decline of cognitive function in AD patients.

